# A Randomized Controlled Open Label Crossover Trial to Study Vaginal Colonization of Orally Administered Lactobacillus Reuteri RC-14 and Rhamnosus GR-1 in Pregnant Women at High Risk for Preterm Labor

**DOI:** 10.3390/nu12041141

**Published:** 2020-04-19

**Authors:** Enav Yefet, Raul Colodner, Merav Strauss, Yifat Gam Ze Letova, Zohar Nachum

**Affiliations:** 1 Department of Obstetrics & Gynecology, Emek Medical Center, Afula 1834111, Israel; yifatkasinetz@hotmail.com (Y.G.Z.L.); nachum.zo@gmail.com (Z.N.); 2 Department of Obstetrics & Gynecology, Baruch Padeh Medical Center Poriya, Tiberias 1410000, Israel; 3 Faculty of Medicine in Galilee, Bar-Ilan University, Safed 1310000, Israel; 4 Microbiology Laboratory, Emek Medical Center, Afula 1834111, Israel; colodner_ra@clalit.org.il (R.C.); meirav_sh@clalit.org.il (M.S.); 5 Rappaport Faculty of Medicine, Technion, Haifa 3200003, Israel

**Keywords:** pregnancy, probiotics, lactobacilli, bacterial vaginosis, preterm delivery, abnormal vaginal flora

## Abstract

Lactobacilli administration has been suggested for the treatment and prevention of bacterial vaginosis, which increases the risk for preterm birth. We aimed to evaluate the vaginal colonization of lactobacilli orally administered to pregnant women at risk for preterm birth. We performed a randomized and controlled crossover study between January 2016 and May 2017. Forty pregnant women at high risk for preterm birth with normal vaginal flora (Nugent score ≤ 3) were randomized to either receive two oral capsules/day each containing 5 × 10^9^ *Lactobacilli* (L.) *rhamnosus* GR-1 and *L. reuteri* RC-14 (*n* = 20) or no treatment (*n* = 20) for 2 months. Treatments were then crossed over for an additional two months. A vaginal examination and swabbing were performed for assessment of bacterial vaginosis at baseline and every month until study completion. At the same time points, vaginal samples were cultured and subjected to matrix-assisted-laser-desorption/ionization-time-of-flight-mass-spectrometry (MALDI TOF-MS) for the detection of the specific bacterial strains contained in the capsules. The primary endpoint was the presence of the administered lactobacilli strains in the vagina during the first two months of follow-up. Thirty-eight women completed the study. During the first two months of treatment, *L. rhamnosus* GR-1 was detected in one (5%) woman on the probiotic treatment and 2 (11%) women receiving no treatment (*p* = 0.6). *L. rhamnosus* GR-1 was detected in vaginal samples of 4 (11%) women during probiotic treatment (of both groups) and *L. reuteri* RC-14 was not detected in any samples. The rest of the endpoints were not different between the groups. Altogether, vaginal colonization of lactobacilli following oral administration is low during pregnancy.

## 1. Introduction

Vaginal infections are common, with bacterial vaginosis (BV) or its milder form, abnormal vaginal flora (AFV), present in 30% of pregnant women [1]. The infection involves replacement of the normal lactobacilli-containing flora by anaerobic bacteria [2] and is diagnosed by using the Nugent criteria. This method forms a standardized method of Gram-stain interpretation, designed to evaluate the vaginal flora to detect BV/AVF. Vaginal swab smears are graded on a 10-point scale based on the presence or absence of *Lactobacillus* morphotypes, gram-variable and gram-negative rods, and curved gram-negative rods. A score of 0–3 indicates a normal flora, a score of 4–6 is representative of AFV, and a score of 7–10 is consistent with BV flora [3,4]. Higher scores of Nugent criteria are given when the amount of *Lactobacillus* in the smear is low.

BV has been shown to be associated with obstetric complications, such as premature rupture of membranes, preterm labor and delivery, chorioamnionitis and post-Caesarean endometritis [5,6]. Antibiotic treatment for BV/AVF is generally the treatment of choice, and was shown to decrease the rate of late abortions and preterm deliveries [7,8,9,10]. However, to date, BV/AVF infection prophylactics are not available, and chronic antibiotic use is not recommended.

Lactobacilli, the most prevalent microorganisms in the vagina, protect it from pathogenic infections by producing antimicrobial compounds (e.g., hydrogen peroxide, lactic acid, bacteriocin-like substances) and by adhering and competing for adhesion sites in the vagina [11]. Oral intake of *Lactobacillus* strains has been shown to improve the microbial pattern in vaginal dysbiosis [12]. Long-term vaginal administration of *Lactobacillus* (L). *rhamnosus*, after oral treatment with metronidazole, to prevent the recurrence of BV, has been shown to effectively maintain a balanced vaginal ecosystem in 96% of patients [13]. In addition, in the non-pregnant population, oral administration of *L. rhamnosus* GR-1 and *L. reuteri* RC-14 effectively restored the vaginal flora [14,15], and *L. acidophilus*-supplemented yogurt was associated with an increased prevalence of colonization of the rectum and vagina by this bacteria [16]. However, the potential of lactobacilli in primary prevention of BV/AVF has never been studied in pregnant women at risk for preterm birth. In addition, oral administration, which is more comfortable, has also not been thoroughly studied.

Exploitation of the oral route to administer probiotics is especially important during pregnancy, when women experience increased vaginal secretions, which might interfere with the ability of the probiotic capsule to adhere adequately to the vaginal mucosa. Concomitant vaginal pregnancy-related medications, such as hormonal supplements, can also contribute to patient discomfort associated with the vaginal route.

The present study evaluated vaginal colonization of lactobacilli orally administered to pregnant women at risk for preterm birth. The effect of lactobacilli administration on primary prevention of vaginal infections was also assessed.

## 2. Subjects and Methods

### 2.1. Design

This open-label, parallel-group, randomized, cross-over and controlled trial was conducted at Emek Medical Center, a university-affiliated hospital in Israel, between January 2016 and May 2017. The study was authorized by the local review board (ID of the approval EMC-96-13) and was registered on www.clinicaltrials.gov (NCT02430246, principal investigator E.Y, registration: 20 April 2015). All participants provided written informed consent. Women above 18 years of age, with risk factors for preterm birth (e.g., previous preterm delivery, multiple pregnancy, preterm uterine contractions and/or cervical effacement/dilation), and with normal vaginal flora (Nugent score of 0–3) were eligible to participate in the study. Women with abnormal vaginal flora or bacterial vaginosis (Nugent score > 3), preterm premature rupture of membranes, immunocompromised, currently on antibiotic treatment, and with an either known or new onset allergy to the study probiotic formula, were excluded from the study. Vaginal candidiasis at baseline was not considered as an exclusion criterion.

Participating women were randomly assigned (1:1) to treatment groups, using a computer randomization sequence generation program. The randomization code was stored in a closed study box in sealed opaque envelopes. The randomization sequence was concealed until intervention was assigned by the study physicians.

### 2.2. Interventions

**Figure 1:**
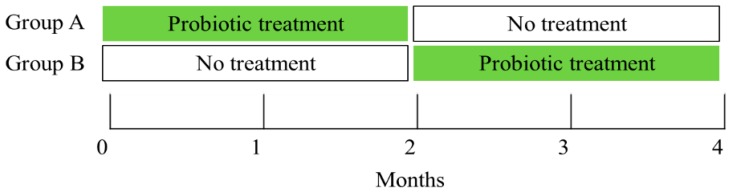
Timetable for schedule of treatment and assessments. At baseline (point 0) and every month until study completion (points 1–4) the following assessment was done: vaginal examination, swabbing for Nugent score, lactobacilli culture, and matrix-assisted-laser-desorption/ionization-time-of-flight-mass-spectrometry (MALDI TOF-MS) for the detection of the specific lactobacilli strains contained in the capsules and for other strains.

At enrollment, study participants were instructed to take no other probiotic products. Group A received two oral capsules/day each containing 5 × 10^9^ *L. rhamnosus* GR-1 and *L. reuteri* RC-14 (CHR. HANSEN, Denmark) for two months, while group B received no treatment for two months. Afterwards, the groups were crossed over and group B received the probiotic capsules while group A received no treatment for an additional two months. *L. rhamnosus* GR-1 and *L. reuteri* RC-14 were selected because they originate from the female urogenital tract, colonize the vagina, and can inhibit the growth and adhesion of urogenital pathogens [17]. Assessing subjective complaints on vaginal symptoms, a per-speculum vaginal examination, pH evaluation with vs. sense (Common sense Ltd., Israel) and pH indicators (SIGMA, ST. Louis, MO, USA), as well as vaginal swabbing for Nugent scores, were performed at baseline and every month, throughout the four-month study period. Vaginal samples, also collected monthly, were cultured for the detection of bacteria contained in the capsules, identified by matrix-assisted laser desorption/ionization time-of-flight mass spectrometry (MALDI TOF-MS; described in details in the following section) following culture on selective media. A diagram of the treatment and assessment schedule is presented in Figure 1.

### 2.3. Laboratory Assessments

All laboratory analyses were performed at the Microbiology Laboratory at Emek Medical Center.

#### 2.3.1. Diagnosis of Vaginal Infections

AVF/BV were diagnosed according to the Nugent criteria [3,4], which were evaluated in vaginal smears taken using a sterile transport swab. A Nugent score > 3 was considered positive for AVF/BV. Candida infection was diagnosed by direct microscopy. The laboratory personnel were blinded to treatment allocations.

#### 2.3.2. Identifying Vaginal Lactobacilli

Vaginal specimens were serially diluted and plated on lactobacillus agar, as previously described [18]. We used a semi-quantitative assessment for lactobacilli growth in culture using a scale of 1 (mild growth) to 4 (extensive growth). Gram staining (Gram-positive rods) and further MALDI TOF-MS analysis of 2–3 independent colonies, as previously described [19,20], were conducted to confirm that the growing bacteria were lactobacilli. This method enables rapid and accurate identification of specific bacterial strains based on physical and electrical characteristics of the bacteria. Briefly, bacteria isolated from Rogosa agar were seeded on the MALDI TOF-MS target plate and allowed to acquire an electrical charge by mixing them with a special matrix. The seeded target was then analyzed in a Maldi Biotyper^®^ (Bruker Daltonics, Bremen, Germany). Briefly, a pulsed laser irradiated the sample, triggering ablation and desorption of the sample and matrix material. Finally, the analyte molecules were accelerated into a mass spectrometer and then analyzed^19^. For the purpose of this study, the lactobacilli from the probiotic capsules were isolated from the capsule and subjected to MALDI TOF-MS to create a specific and highly accurate profile of the sub-species ratios in the administered capsules. A score of ≥1.9 in the MALDI Biotyper was considered as an accurate identification of the sub-species level. This database was then used to identify the bacteria in the vaginal samples obtained from all study participants. MALDI-TOF was also used to identify native vaginal lactobacilli.

### 2.4. Study Endpoints

The primary endpoint was the presence of either *L. rhamnosus* GR-1 or *L. reuteri* RC-14 in at least one of the two culture swabs taken during the first two months. Rate of vaginal infections and vaginal pathologic findings were also evaluated.

In addition, the study was designed as a crossover study in order to evaluate the duration of colonization of the specific lactobacilli from the capsules after treatment discontinuation in group A, and also to evaluate their natural appearance in the vagina during the first 2 months of no treatment in group B. The presence of the lactobacilli during probiotic treatment (first two months of group A and second two months of group B) was also assessed. Compliance and adverse effects were assessed at each study visit, by directly questioning patients about specific possible adverse effects (e.g., diarrhea, vomiting, abdominal pain, etc.) and also by posing open questions. In addition, emergency ward visits and hospitalizations were recorded and data from the medical charts were collected.

### 2.5. Sample Size and Statistical Analysis

Assuming a 75% and 25% detection rate of the lactobacilli from the probiotic capsules during the first two months of follow-up in the treatment (group A) and no-treatment (group B) groups, respectively, 15 women were required for each group (+5 women per group for cases of drop out, 5% 2-sided alpha, 80% power). Groups’ subject characteristics and endpoints were compared using Student’s *t*-test (or the Wilcoxon two sample test) for continuous variables and χ2 (or Fisher’s exact test) for categorical variables. Analysis was performed according to the intent-to-treat principle. The statistical analyses were performed using SAS 9.4 software (SAS Institute Inc., Cary, NC, USA.). Statistical significance was determined when *p* < 0.05.

## 3. Results

**Figure 2:**
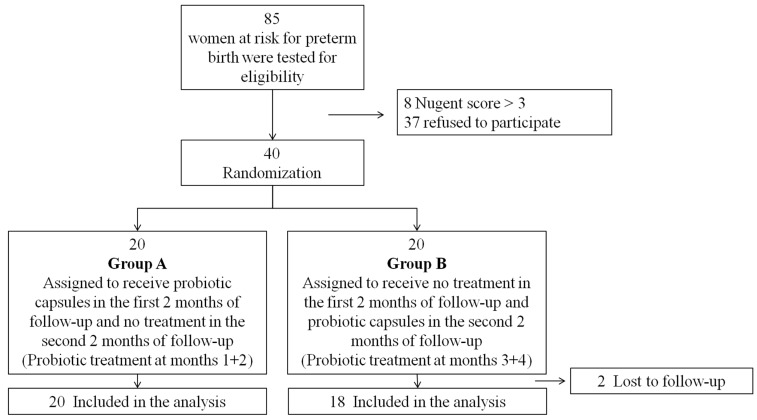
Patients’ flow chart.

Of 77 women who met the inclusion criteria, 40 women (52%) agreed to participate in the study (Figure 2). Two women in group B were lost to follow-up. All other women were included in the analysis. Gestational week at recruitment was higher and history of preterm birth as a risk factor for preterm birth was more prevalent in group A as compared to group B. All other baseline characteristics were similar across the two groups (Table 1).

**Figure 3:**
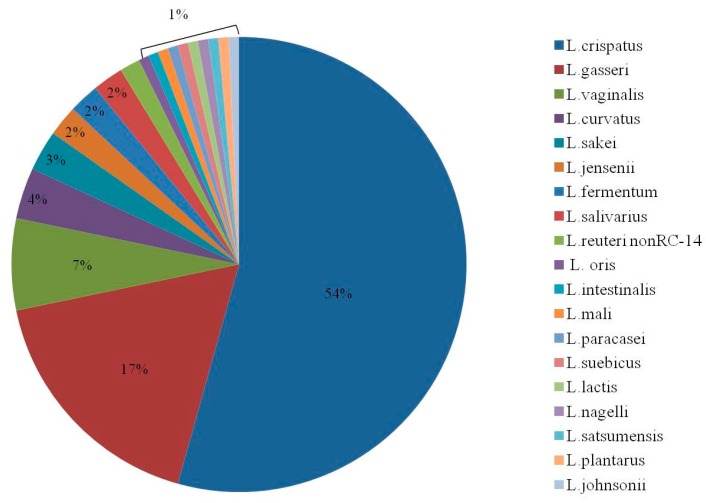
Percentage of native lactobacilli in vaginal specimens.

Study endpoints are described in Table 2. *L. reuteri* RC-14 was not detected in vaginal specimens. Data from follow-up months 3 + 4 were missing for four women from group A, who were therefore excluded from this analysis. Six women presented *L. rhamnosus* GR-1-positive vaginal samples, 4 of whom showed *L. rhamnosus* GR-1 colonization during probiotic treatment. Two women from group B had *L. rhamnosus* GR-1-positive vaginal samples during the first two months of the study, when probiotics were not given. Following questioning, they reported that they took probiotic products out of the study protocols. One woman took probiotic capsules which contained *L. rhamnosus* GR-1. The other woman ate a non-commercial yogurt enriched with lactobacilli, and information regarding the exact lactobacilli composition was not available. *L. rhamnosus* GR-1 disappeared after the external probiotic discontinuation. The incidence of probiotics lactobacilli-positive samples during the first two months of treatment was similar between the two groups (one (5%) woman in group A versus two (11%) women in group B; *p* = 0.6). Additionally, one woman in group B had L. *rhamnosus* GR-1 in months 3 + 4 (which were not included in the primary outcome). The growth of *L. rhamnosus* GR-1 in all specimens reached only 1–2 points in a scale of 1–4. In addition, each specimen had one or two native lactobacilli strains. The percentage of the native vaginal lactobacilli strains is presented in Figure 3. The most prevalent lactobacilli were *L*. *crispatus* (54%), *L. gasseri* (17%), and *L. vaginalis* (7%).

Five women had vaginal infection during the study (Table 2). One woman in group A had vaginal discharge consistent with candidiasis at baseline, and after the first and second months in vaginal examinations. She was treated with vaginal tablets of clotrimazole. Another woman in group A suffered from vaginal discharge consistent with candidiasis after two months of treatment, and was also treated with a vaginal tablet of clotrimazole. Three women in group B experienced bacterial vaginosis (two after two months of follow-up and one after three months of follow-up). They were offered an antibiotic treatment with either metronidazole or clindamycin, but all refused treatment. The prevalence of vaginal infections during the first months of follow-up was not different between the groups (*p* = 0.6).

No other endpoints, including the rate of vaginal pathologic findings and the effect of crossover on the probiotic-formula lactobacilli colonization dynamics, were statistically different between the groups (Table 2). No adverse events related to the study treatment were reported.

## 4. Discussion

In the present study, we evaluated the vaginal colonization efficacy of orally delivered lactobacilli, as well as its potential as a vaginal infection prophylactic in women at risk for preterm birth. We found that after two months of treatment, only *L. rhamnosus* GR-1 but not *L. reuteri* RC-14 colonized the vagina, albeit, at a very low rate. Moreover, vaginal infection rate was low and was not impacted by lactobacilli treatment. However, due to the low infection rate, definitive conclusions cannot be drawn, studies with a larger sample sizes and longer durations of treatment will be necessary to further assess this aspect. Group A, which was treated in the first two months, had a higher rate of past preterm births as compared to group B. Since both groups were eventually treated with probiotics for two months, we do not believe that this difference had a significant effect on the results.

Lactobacilli have been previously demonstrated to confer protection against vaginal infections. The mode of action is likely multi-fold, including prevention of bacterial ascending from the rectum to the vagina, reduced ascension of pathogens, or an immunomodulatory effect [11]. During pregnancy, *L. iner* alone was detected in 85% of women who delivered preterm and only in 16% of women who delivered at term [21]. Both *L. rhamnosus* GR-1 and *L. reuteri* RC-14 have been shown to colonize the vagina after their vaginal [22] or oral administration [14,15] to non-pregnant women. It was hypothesized that the gut may function as a reservoir for lactobacilli that colonize the vagina [23,24]. In the present study, the rate of colonization was much lower than previously reported, even though colonization following oral administration was witnessed when probiotics were given for a similar duration of treatment [14]. In parallel, the latter study showed that administration of lactobacilli for 60 days provided primary protection against BV, as shown by the statistically significant difference of a 24% infection rate among placebo-treated women who did not have a BV infection at baseline, as compared to 0% infection rate in the lactobacilli-treated group [14]. There are several possible explanations for the discrepancy between the two studies. Firstly, possible slowing of the gastrointestinal system due to pregnancy-related hormonal changes, which subsequently slows the transfer of lactobacilli from the intestinal track. In such a case, a longer period of time might be necessary to demonstrate vaginal colonization. Secondly, in the study involving non-pregnant women, 25% of the women had an active BV infection at recruitment, while in the present study, only woman with normal vaginal flora were eligible to participate. It is possible that in the presence of BV, lactobacilli better colonize the vagina than when the vagina is colonized with other native lactobacilli. Thirdly, in the previous study, the total amount of vaginal lactobacilli was tested, rather than the presence of the specific bacterial strains administered. Finally, it is possible that oral probiotic administration affects the vagina through indirect mechanisms, such as immunomodulation or competitive inhibition of pathogens in the gastrointestinal tract, and not by direct colonization. Since in non-pregnant populations both *L*. *Rhamnosus* and *L*. *reuteri* species were able to undergo gastric passage [25] and colonize the vagina [14,15] future studies should focus on elucidating the ability of successful gastric passage of those lactobacilli during pregnancy.

To our knowledge, this is the first study to use MALDI TOF-MS to detect the presence of specific lactobacilli strains. It should be noted that before and during the study, MALDI TOF-MS successfully detected the lactobacilli in the capsules, thus, mal-production of the capsules as the reason for ineffectiveness was ruled out. This technique has also been previously demonstrated to be useful in detecting specific probiotic strains in food and yoghurts [20], as well as bacteria and fungi in samples from various origins including blood, urine, and cerebrospinal fluids [19,26,27]. More specifically, MALDI TOF-MS successfully identified specific lactobacilli strains in vaginal specimens [28] and Group B *Streptococcus* (GBS) in vaginal and rectal specimens that were collected from pregnant women [29]. It is possible that the amount of lactobacilli transferred to the vagina was too small to be detected, particularly when they were grown together with other lactobacilli strains from the vagina. 

The strengths of this study were its randomized controlled design, as it was the first of its kind to examine the ability of orally administered probiotics to colonize the vagina of pregnant women at risk for preterm birth and the high compliance rate. The limitations of the study were that obstetrical outcomes were not addressed, due to the small sample size and short duration of treatment. Moreover, although participants were instructed to abstain from using any other probiotic product, two women took probiotic products, contrary to the guidelines. Retrospective assessment of probiotic consumption was partial, as we did not have available data regarding the composition of the probiotic strains that were taken by one of the participants. In addition, the rate of preterm birth was not assessed, and this should be done in future trials.

## 5. Conclusions

Altogether, the rate of vaginal lactobacilli colonization after their oral administration over two months was low in pregnant women with a normal vaginal flora and at risk for preterm birth. The effect of oral probiotic administered over longer periods and on treating vaginal infections should be examined in future studies.

## Figures and Tables

**Table 1:** Baseline characteristics of study participants.

	Group A (Probiotic Treatment at 1 + 2 Months) *n* = 20	Group B (Probiotic Treatment at 3 + 4 Months) *n* = 18	*p* Value
Age	32.1 (5.0) [32.0]	31.8 (4.0) [32.5]	0.9
Body mass index	24.0 (3.9) [23.5]	23.7 (4.6) [22.2]	0.65
Previous pregnancies	3.3 (1.3) [3]	3.9 (2.2) [3]	0.49
Number of children	1.7 (1.0) [2]	1.8 (1.3) [2]	0.8
Previous abortions	0.6 (0.8) [0]	1.2 (1.5) [1]	0.18
Previous births	1.8 (1.1) [2.0]	1.8 (1.4) [1.5]	0.89
Gestational week	19.4 (4.1) [19.2]	16.3 (3.5) [17.2]	0.02
Subjective vaginal complaints	2 (10%)	2 (11%)	1
Number of women with findings in per-speculum (PS) examination	2 (10%)	1 (6%)	1
pH > 5	1 (5%)	0 (0%)	1
Positive vs. sense	1 (5%)	0 (0%)	1
Risk Factors for Preterm Labor *			
Number of previous preterm deliveries	0.8 (0.6) [1]	0.3 (0.5) [0]	0.02
Preterm uterine contractions	1 (5%)	1 (6%)	1
Cervical shortening	2 (10%)	1 (6%)	1
Bleeding from placenta previa	0 (0%)	1 (6%)	0.47
Past abruption	0 (0%)	1 (6%)	0.47
Malformed uterus	1 (5%)	0 (0%)	1
Past preterm delivery	14 (70%)	6 (33%)	0.02
Past late abortion **	0 (0%)	3 (17%)	0.1
Multiple pregnancy	4 (20%)	8 (44%)	0.1

Values are presented as mean (SD) [median] or number (percent). * If several risk factors co-existed, they are written in all the appropriate rows. ** At gestational weeks 21–16.

**Table 2:** Study endpoints.

	Group A (Probiotic Treatment at 1 + 2 Months) *n* = 20	Group B (Probiotic Treatment at 3 + 4 Months) *n* = 18	*p* Value
Compliance with study protocol ^$^			
Full	20 (100%)	16 (80%)	
Partial	0 (0%)	2 (20%)	0.11
Subjective vaginal complaints			
In the first 2 months of treatment *	3 (15%)	0 (0%)	0.23
In the second 2 months of treatment *^€^	2 (13%)	1 (6%)	0.59
Findings in per-speculum (PS) examination			
In the first 2 months of treatment *	2 (10%)	0 (0%)	0.49
In the second 2 months of treatment *^€^	1 (6%)	0 (0%)	0.47
Presence of *L. rhamnosus* GR-1			
In the first 2 months of treatment *	1 (5%)	2 (11%)	0.6
In the second 2 months of treatment *^€^	0 (0%)	3 (17%)	0.23
Vaginal infection ^§^			
In the first 2 months of treatment *	2 (10%)	2 (11%)	1
In the second 2 months of treatment *^€^	0 (0%)	1 (6%)	1
Effect of crossover on *L. rhamnosus* GR-1 colonization ^€^			
None	16 (100%)	13 (72%)	0.08
lactobacili appeared	0 (0%)	3 (17%)	
lactobacili disappeared	0 (0%)	2 (11%)	
Effect of crossover on vaginal infection ^€^			
None	15 (94%)	15 (83%)	1
Vaginal infection resolved	1 (6%)	2 (11%)	
Vaginal infection developed	0 (0%)	1 (6%)	

Values are presented as number (percent). ^$^ By self report. * In at least one out of the two visits. ^€^ 4 women from Group A had no information on months 3 + 4 of follow-up and were therefore excluded from this analysis. ^§^ Vaginal infection refers to abnormal vaginal flora, bacterial vaginosis, and candidiasis.
